# EMAP-II-dependent lymphocyte killing is associated with hypoxia in colorectal cancer

**DOI:** 10.1038/sj.bjc.6603299

**Published:** 2006-08-22

**Authors:** M M S Youssef, P Symonds, I O Ellis, J C Murray

**Affiliations:** 1 Wolfson Digestive Diseases Centre, University Hospital, Nottingham NG7 2UH, UK; 2 Division of Pathology, School of Molecular Medical Sciences, University of Nottingham, Nottingham, UK

**Keywords:** EMAP-II, hypoxia, lymphocytes apoptosis, colorectal cancer

## Abstract

Endothelial-monocyte-activating polypeptide-II (EMAP-II) is a novel multifunctional polypeptide with proinflammatory activity. We have previously shown that the recombinant and native forms of EMAP-II can induce apoptosis in mitogen-stimulated lymphocytes, and that the release of this protein into the extracellular milieu is enhanced by hypoxia. We hypothesised that hypoxia may lead to death of tumour-infiltrating lymphocytes (TILs) via an EMAP-II-dependent mechanism, thereby assisting tumours to evade the immune system. In this study, we used immunohistochemistry to detect EMAP-II, active caspase-3 and cleaved Poly (ADP-ribose) Polymerase (PARP) as indicators of apoptosis in TILs, and carbonic anhydrase IX (CA IX) as a surrogate marker of hypoxia. EMAP-II expression is associated with regions of hypoxia, and furthermore there is a significant association between TILs apoptosis and the presence of hypoxia. Using a coculture model of colorectal cancer cell/lymphocyte interactions, we were also able to demonstrate lymphocyte apoptosis induced by tumour cells, with concomitant caspase-3 activity. Lymphocyte killing was enhanced by direct cell–cell contact, particularly by tumour cells exposed to hypoxic conditions. Our data support the hypothesis that hypoxia plays a role in immune evasion by tumour cells, through EMAP-II-dependent lymphocyte killing.

Endothelial-monocyte-activating polypeptide-II (EMAP-II) was first detected in supernatants of cultured murine tumour cells (Berger *et al*, 2000; Murray and Tas, 2001) based on its ability to stimulate procoagulant activity in cultured endothelial cells. Kao *et al* (1992) isolated 20–22 kDa EMAP-II protein, which proved to have pleiotropic, cytokine-like activity towards endothelial cells as well as monocytes and neutrophils (Kao *et al*, 1994). EMAP-II is believed to be synthesised as a 34-kDa precursor molecule, which is proteolytically cleaved to produce the 20-kDa mature polypeptide (Tas *et al*, 1997). EMAP-II and the p43 auxiliary component of the mammalian multisynthetase complex share a high degree of amino-acid identity (Quevillon *et al*, 1997), although the precise relationship between these polypeptides is not understood.

It has been suggested that EMAP-II is released from cells as a consequence of activation of programmed cell death (Knies *et al*, 1998), and we showed that enhanced processing and release of mature protein are initiated in response to cellular stress (Barnett *et al*, 2000). More recently, we showed that EMAP-II induces apoptosis in mitogen stimulated lymphocytes, and suggested that EMAP-II might therefore act in an immuno-suppressive role in the tumour milieu, protecting tumour cells from activated lymphocytes (Murray *et al*, 2004).

Colorectal cancer (CRC) is characterised by regions of variable hypoxia (Moulder and Rockwell, 1984). Hypoxic growth can result in a tumour with more aggressive growth characteristics and more malignant phenotype (Harris, 2002). Hypoxia-induced apoptosis of tumour-infiltrating lymphocytes (TILs) might provide another growth benefit to the tumour, favouring growth of more resistant tumour cells. In this study, we provide evidence to support the hypothesis that hypoxia leads to apoptosis of TILs, and that EMAP-II may play a role in this mechanism, protecting tumour cells against the immune system.

## MATERIALS AND METHODS

### Tissue samples

Formalin-fixed, paraffin-embedded archival tissue samples from 72 patients diagnosed with colorectal tumours at the Nottingham City Hospital between 1991 and 1992 were used in this study. All tumour samples were coded to conserve patient confidentiality. The adenomatous lesions included one villous, four tubulo-villous and six tubular tumours. The study was approved by the Research Ethics Committee of Nottingham City Hospital.

### Cell lines

The human leukemic T-cell line Jurkat, the colorectal adenocarcinoma cell lines DLD-1 and HT29 were obtained from American Type Culture Collection (Manassas, VA, USA).

### Cell culture and induction of hypoxia *in vitro*

The cell lines were cultured in RPMI-1640 medium (Life Technologies, Paisley, UK), supplemented with 10% fetal calf serum (PAA Laboratories, Lintz, Austria) and 100 U ml^−1^ penicillin/streptomycin solution (Sigma-Aldrich, Poole, UK). Cells were maintained at 37°C in 5% CO_2_ in a humidified incubator and were routinely subcultured by removal from flasks with 0.05% trypsin/1 mM EDTA (Sigma-Aldrich). For exposure to a hypoxic environment, subconfluent cells in serum-free medium were incubated in a hypoxic chamber containing 1% O_2_, 5% CO_2_, and 94% N_2_ for 4, 16 and 24 h and in normal conditions as a control.

### Coculture model

The contact and noncontact cocultivations were carried out in 24-well plates (5 *μ*m pore size; Corning Costar, Cambridge, UK). For noncontact coculture, DLD-1 and HT29 tumour cells were added at 50 000 cells well^−1^ in the lower chambers. We kept on looking at the cells to assure that they adhered to the wells. On the day of the experiment, we treated some cells either by tumour necrosis factor-*α* (TNF-*α*) and interferon-*γ* (IFN-*γ*) 20 ng ml^−1^ to enhance release of EMAP-II or with TNF-*α*/IFN-*γ* and rabbit polyclonal antibodies against human EMAP-II (R2B2) at different concentrations to block endogenous EMAP-II. Purified rabbit IgG (IgG) antibody (R&D Systems, Abingdon, UK) was used as a negative control. The Jurkat cells were plated out at. The Jurkat cells were plated out at 20 0000 cells well^−1^ in the upper chamber in the presence of phytohaemag-glutinin (PHA) (1 *μ*g ml^−1^). In contact coculture, there is a coculture in the same well. The culture was done in normoxia and hypoxia for 4 and 16 h, respectively. The nonadherent Jurkats were removed and centrifuged at 1000 × g for 5 min. Samples were analysed using active caspase-3, described below. The samples were done in triplicates.

### Caspase-3 activity assay

A commercially available kit was used to detect activated caspase-3 activity in cell lysates (Oncogene, Research Products, Nottingham, UK), which takes advantage of the specificity of the enzyme for cleavage after aspartate residues in particular peptide sequence (DEVD). The DEVD substrate is labelled with a fluorescent molecule, 7-amino-4-trifluoromethyl coumarin (AFC), and the reaction is monitored by a blue to green shift in fluorescence upon cleavage of the AFC fluorophore. Caspase-3 activity was measured in extracts of control Jurkat T cells, cells from contact or noncontact cocultures with tumour cells in normoxia and hypoxia after 4 and 16 h, respectively, using a fluorescent plate reader. The data are expressed as the relative fluorescence intensity (RFU) after subtraction of the relative signal of the appropriate buffer controls. RFU represents sample fluorescence/control fluorescence intensity based on three separate experiments.

### Assessment of apoptosis by FITC-labelled annexin-V iodide

Annexin-V and propidium iodide (PI) reagents can be used together to distinguish early and late apoptosis (Vermes *et al*, 1995; Martin *et al*, 1995). Jurkat T cells, following coculture with tumour cell monolayers for 24 h under normal and hypoxic conditions, were assessed for apoptosis. Tumour cells were either untreated or treated with 20 ng ml^−1^ TNF-*α*/IFN-*γ*, and in the presence or absence of R2B2 antibodies. Purified normal rabbit IgG was used as a control (R&D Systems). Samples were analysed using the Apotest-FITC kit (DAKO, Glostrup, Denmark), in accordance with the manufacturer's instructions. Median percentage of apoptotic Jurkat cells was detected. The data are the averages of three experiments.

### Detection of EMAP-II by Western blotting

PAGE was used to separate proteins from DLD-1 and HT29 cell extracts and supernatants from coculture experiments for 4 and 24 h. EMAP-II was detected by Western blotting with the R2B2 polyclonal antibodies as described previously (Tas *et al*, 1997; Murray *et al*, 2004). Antibody binding was revealed by enhanced chemiluminescence.

### Enzyme-linked-immuno-sorbent assay for soluble EMAP-II

Detection of soluble EMAP-II was performed after incubating cells in serum-free RPMI medium for four and 24 h in normal and hypoxic conditions in coculture experiments. Recombinant EMAP-II was dissolved in RPMI medium to a final concentration of 50 ng ml^−1^ for use as external standard. Samples were analysed using human EMAP-II (Bio-Source International, Camarillo, CA, USA).

### Flow cytometric analysis of cell surface expression of EMAP-II

DLD-1 and HT29 cells were incubated in RPMI medium for 24 h in normal and hypoxic conditions. Immunofluorescent detection of EMAP-II was performed as described previously (Murray *et al*, 2004). Staining was carried out on fixed, permeabilised cell suspensions with R2B2 antibodies or purified IgG from preimmune rabbit serum (R&D Systems) at 1 *μ*g ml^−1^ for 2 h. The secondary antibody was goat anti-rabbit FITC-conjugate (Sigma-Aldrich). Additional cell samples were incubated with the secondary FITC-conjugated antibody, but without primary antibody, as controls. Samples were analysed on a Becton Dickinson FACScan using the LYSIS program.

### Antibodies and recombinant proteins

EMAP-II was detected using the polyclonal rabbit antibody R2B2. The characteristics of R2B2 have been described elsewhere (Tas *et al*, 1997; Barnett *et al*, 2000; Murray *et al*, 2000). HRP- and FITC-labelled anti-rabbit or anti-goat IgG antibodies (Sigma-Aldrich) were used for detection of primary antibodies. Recombinant Human carbonic anhydrase IX (CA IX) is a *trans*-membrane glycoprotein, first sequenced and characterised as membranous CA IX (MN/CA IX) by Opavsky *et al* (1996), and is recognised as a surrogate marker of hypoxia in tumours (Beasley *et al*, 2001; Wykof *et al*, 2001). Polyclonal anti-CA IX antibody antibody was a gift from Bayer Pharma (Germany). Rabbit polyclonal antibody against active caspase-3 was obtained from R&D Systems (Abingdon, UK). Cleaved Poly (ADP-ribose) Polymerase (PARP) antibody was obtained from Cell Signaling (UK). Recombinant human TNF-*α* and IFN-*γ* were purchased from PeproTech (London, UK).

### Immunohistochemistry

Archival samples of colorectal cancers were analysed for EMAP-II, CA IX, active caspase-3, and cleaved PARP expression. Slides were dewaxed in Histolene (Cell Path Plc, Hemel Hempstead, UK), before being rehydrated in graded ethanol solutions (100–30%). Antigen retrieval was performed by boiling the slides for 10 min in citrate buffer (10 mM citric acid, 25 mM sodium hydroxide). Slides were blocked with normal goat serum for 20 min. EMAP-II and CA IX were identified by incubating the slides with purified polyclonal antibodies R2B2 (1 *μ*g ml^−1^ in PBS), M75 anti-human CA IX antibody at a 1 : 250 dilution for 1 h at room temperature, respectively. Secondary detection was performed using the Vectastain Elite kit, according to manufacturer's instructions (Vector Laboratories, Burlingame, CA, USA). Slides were counterstained with haematoxylin solution (Vector Laboratories), dehydrated in ethanol, and mounted with DePeX polystyrene solution (BDH, Poole, UK).

Apoptotic cell death was detected in TILs by immunohistochemistry using antibodies against active caspase-3 (R&D Systems) and cleaved PARP antibody (Cell Signaling). After deparaffinisation and rehydration, an antigen retrieval was performed, as described above. Active caspase-3 was identified by incubating the slides with rabbit polyclonal against active caspase-3 (1 : 200 in TBS) for 1 h at room temperature. Cleaved PARP was detected by incubating the slides with cleaved PARP antibody at a 1 : 50 dilution in TBS at 4°C overnight. Secondary detection was performed as described above.

### By-eye evaluation of active caspase-3 and cleaved PARP staining

Cells were identified as active caspase-3-positive if there was cytoplasmic and perinuclear localisation of stain. Localisation of stain was nuclear in cleaved PARP-positive cells. From five randomly selected fields of each section, two independent observers, blinded to the clinicopathological features of these cancers, counted one thousand cells. According to the proportion of positive cells, the degree of staining achieved with their antibodies was graded as follows: negative (−); +, 1–25% of cells positive; ++, 26–50% of cells positive; +++, 51–75% of cells positive; ++++, 76–100% of cells positive.

### Image analysis

Quantitative data for EMAP-II and CA IX expression were acquired with a semiautomatic method based on computerised digital image analysis system (Gray Cancer Institute, UK), which has been shown to be a reproducible method (Araya *et al*, 1998; de Moissac *et al*, 2000). Analysis of the hypoxic marker and EMAP-II was performed with × 100 magnification. A contour line was drawn to delineate the area of EMAP-II and CA IX expression in the tumour area. Four different sections of each slide were analysed for EMAP-II and CA IX staining; the parameters measured were (1) tumour area in the field of view, (2) the tumour area stained for each protein and (3) the stained area as a percentage of the tumour area. The mean of these parameters was used for each patient in the statistical analysis. Analysis settings were reviewed and confirmed by Dr JC Murray.

### Statistical analysis

Analysis of activity of caspase-3 and median percentage of apoptotic cells was performed with Microsoft Excel (Redmond, WA, USA). The data were expressed as mean±s.e.m. from three independent experiments and the statistical significance was tested by the Student's test. Statistical analysis was performed using the SPSS for Windows program package (Chicago, IL, USA). For two variables measured on a continuous scale, the Spearman rank correlation test was used to test the correlation. Multivariate regression analysis was performed including different pathological features. *P*-values ⩽ 0.05 were considered significant. Analysis of ELISA data was performed with Microsoft Excel (Redmond, WA, USA).

## RESULTS

### Apoptosis of tumour-infiltrating lymphocytes induced by hypoxia in colon cancer

**Figure 1:**
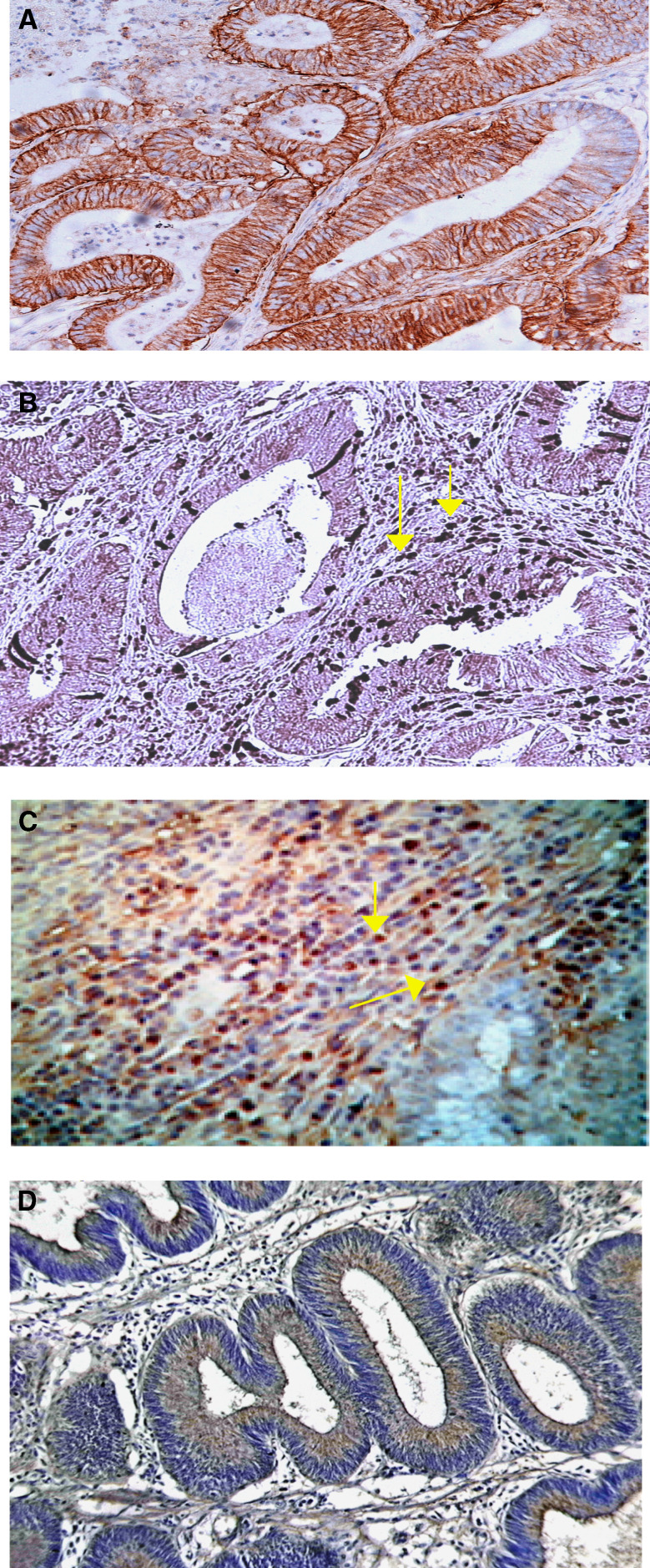
Association between hypoxia, TILs apoptosis and EMAP-II in CRC *in vivo*. (**A**) Immunohistochemical staining of a moderately differentiated adenocarcinoma of the colon with polyclonal anti-CA IX antibody. There is a strong membranous and cytoplasmic staining within the malignant epithelial cells (original magnification × 200). (**B**) TILs in tumourous tissues were strongly reactive with antiactive caspase-3 antibody (original magnification × 400). (**C**) Apoptotic TILs immunostained against cleaved PARP antibody (original magnification × 400). (**D**) Strong brown cytoplasmic staining of malignant epithelial cells in the tumour with EMAP-II (original magnification × 200).

As a surrogate marker for detection of hypoxia, we examined expression of CA IX in archival specimens of colorectal tissues by immunohistochemistry. To evaluate apoptosis of TILs in hypoxic regions, we detected the presence of apoptotic cells using active caspase-3 and cleaved PARP. Figure 1A shows the typical pattern of a section of colorectal carcinoma, with staining of CA IX. There is a strong membranous and cytoplasmic staining within the malignant epithelial cells. Weak staining of CA IX was observed in 16 carcinomas (22%), whereas 27 (37%) showed a moderate positive reaction and 29 (41%) a strong reaction. The 11 adenomatous lesions were obtained from 11 patients. No staining for CA IX was found in one lesion (10%) whereas seven lesions showed weak staining (63%) and three (27%) a moderate reaction. The pattern of apoptotic TILs was observed through areas of hypoxic tumour. Figure 1B shows moderate reactivity to active caspase-3 in TILs. Figure 1C shows immunohistochemical staining of cleaved PARP antibody detecting the nuclear localisation of cleaved PARP in TILs surrounding the colorectal tumour. One apoptotic lymphocyte was detected in TILs in normal colon (Supplementary Figure 1).

### Relationship between CA IX, cleaved PARP, and caspase-3 expression and the clinicopathological features of colorectal cancer

Increased levels of CA IX expression was significantly correlated with presence of metastasis (*P*=0.01); increasing Dukes' stage (*P*<0.01); lymphatic metastasis (*P*=0.01); vascular metastasis (*P*=0.05); lymph node metastasis (*P*=0.02); death (*P*=0.03) and recurrence (*P*=0.02), but not with tumour size, site, differentiation, gender or age of patients (*P*>0.05) (Table 1). Patient and tumour characteristics details are given in supplementary Table 1. In comparison, cleaved PARP in TILs showed significant correlation with lymphatic metastasis (*P*=0.04) and increasing Dukes' stage (*P*=0.02), although not with other features (*P*>0.05) (Table 2). Caspase-3 expression in TILs showed significant correlation with increasing Dukes' stage (*P*=0.02), but not with other features (*P*>0.05) (Supplementary Table 2).

### Colorectal cancer cells express high levels of EMAP-II in apoptotic regions

We examined expression of EMAP-II in archival specimens of colorectal tissues by immunohistochemistry. Figure 1D shows a moderately differentiated adenocarcinoma of the colon stained with EMAP-II. There is strong cytoplasmic staining within the malignant epithelial cells. Weak staining for EMAP-II was found in seven carcinomas (10%), whereas 14 (20%) showed a moderate positive reaction and 51 (70%) a strong reaction. Most adenomatous lesions showed a weak or moderate staining for EMAP-II.

### Relationship between EMAP-II expression and the clinicopathological features of colorectal cancer

Increased levels of EMAP-II expression was significantly correlated with presence of metastasis (*P*=0.05); increasing Dukes' stage (*P*<0.01); presence of lymphatic metastasis (*P*=0.05); vascular metastasis (*P*=0.01); lymph node metastasis (*P*<0.01); death (*P*=0.03) and development of recurrence (*P*=0.01). No associations with other clinicopathological features were found (*P*>0.05) (Table 3).

### Association between hypoxia, TILs apoptosis and EMAP-II in colorectal cancer

Interestingly, our results showed CA IX expression in colorectal cancer was positively associated with cleaved PARP expression in TILs (*P*=0.02), as well as with active caspase-3 expression in TILs (*P*=0.04). EMAP-II expression was also correlated with CA IX expression in CRC patients (*P*=0.03).

### EMAP-II is released under hypoxic conditions *in vitro*

**Figure 2:**
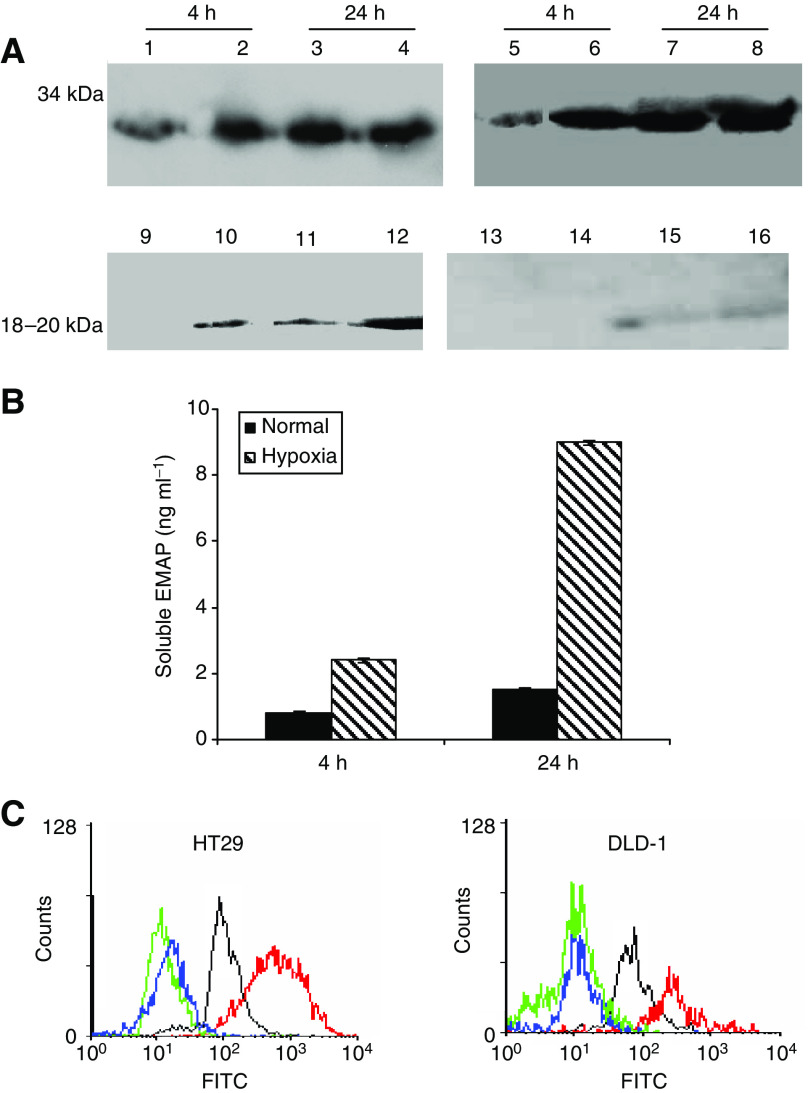
Effect of hypoxia on EMAP-II expression in CRC *in vitro*. (**A**) Western blot of extracts of DLD-1 (Lanes 1–4 and 9–12) and HT29 cells (Lanes 5–8 and 13–16). Cells were grown in control or hypoxia for 4 and 24 h. Lanes 1, 3, 5, 7, control cell lysates at 4, 24 h; Lanes 9, 11, 13, 15 conditioned media from the same cells; Lanes 2, 4, 6, 8, lysate from hypoxic cells for 4, 24 h; Lanes 10, 12, 14, 16 conditioned media from hypoxic cells. (**B**) ELISA of conditioned medium from DLD-1 for soluble EMAP-II. Cells exposed to hypoxia for 4 and 24 h. Data represent mean of three determinations±s.d. (**C**) Flow cytometric determinations of cell surface EMAP-II expression in hypoxia. Untreated DLD-1 and HT29 tumour cells without primary antibody (dashed lines); untreated cells incubated with preimmune antibodies (dotted lines); control cells incubated with R2B2 antibodies against EMAP-II (thin lines); hypoxic cells incubated with R2B2 antibodies against EMAP-II (thick black lines).

Our results suggest that hypoxia induces apoptosis of lymphocytes through EMAP-II, to confirm that EMAP-II are expressed by hypoxic tumour cells, we examined protein expression of EMAP-II by western blotting, flow cytometry and ELISA for soluble EMAP-II. Figure 2A demonstrates that mature EMAP-II protein was detected in the supernatants of DLD-1 cells exposed to hypoxia for four and 24 h. Conditioned medium from HT29 cells contain barely detectable levels of soluble EMAP-II in normal and hypoxic conditions. The blots show a relative increase in *M*_*r*_ 34 000 EMAP-II in hypoxic cell lysates.

There was significant change in total EMAP-II antigen in hypoxic cultures by ELISA (Figure 2B). Consistent with the Western blotting data, exposure to hypoxia for 24 h resulted in a four-fold increase in release of EMAP-II by DLD-1. We observed significant difference in EMAP-II expression between normal and hypoxic cells at four and 24 h. Conditioned media derived from the cultures of HT29 cells showed barely detectable levels in normal and hypoxic conditions (Supplementary Figure 2).

Cell surface expression of EMAP-II on colorectal cancer cell lines was examined by flow cytometry using R2B2 polyclonal antibodies (Figure 2C). Both DLD-1 and HT29 demonstrated cell surface expression of EMAP-II, showing increases in mean fluorescence over negative controls in the range of three- to four-fold. The specificity of this staining was confirmed by the use of a preimmune antibody control, which showed no membrane staining in any of the cell lines. Hypoxia caused a further seven-fold increase in mean fluorescence associated with R2B2 antibody binding to HT29 cells. There is only two- to three-fold increase in hypoxic DLD-1 cells.

### Apoptosis of T-cells induced by hypoxia is EMAP-II-dependent *in vitro*

**Figure 3:**
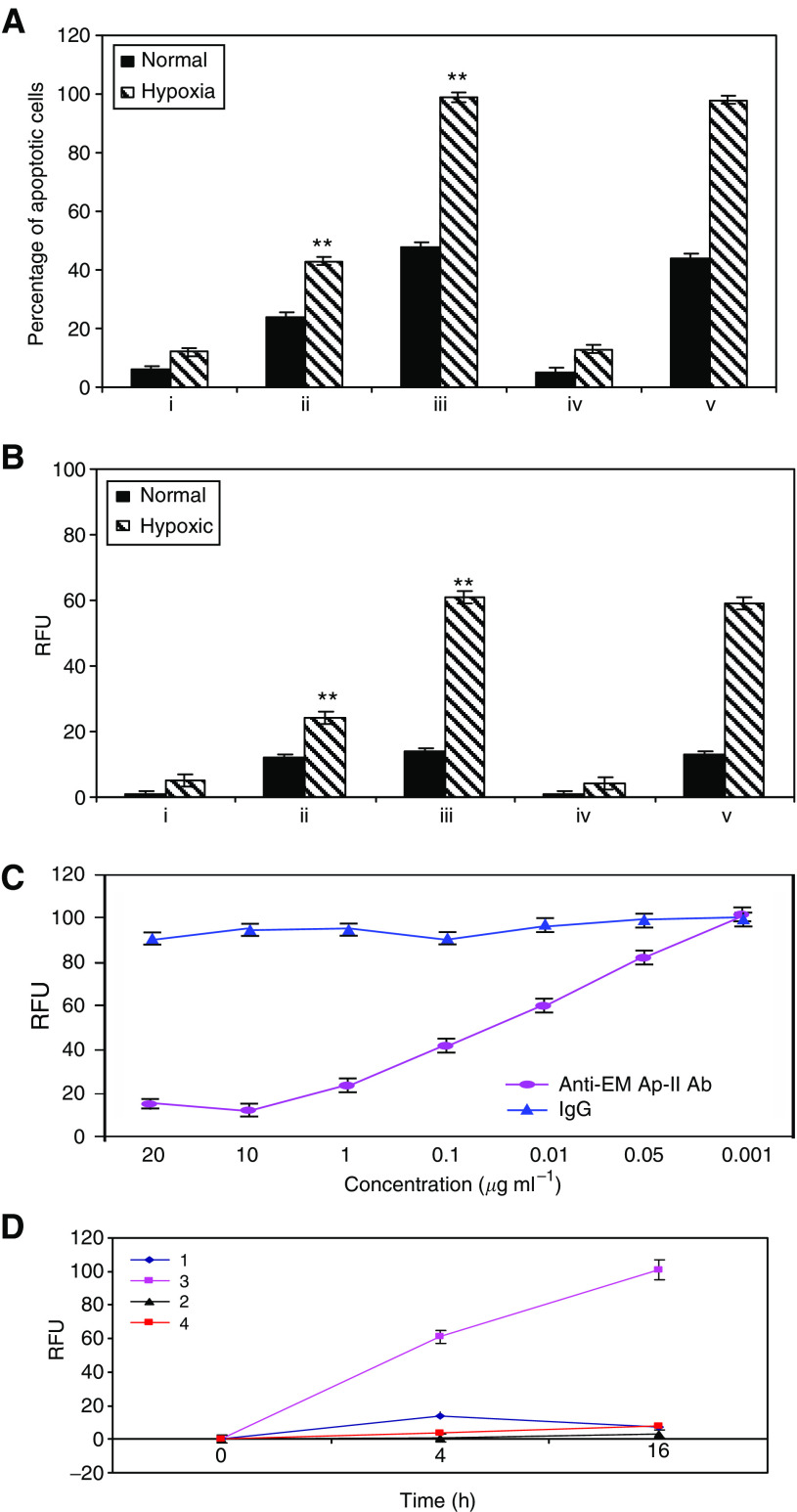
Hypoxia induced apoptosis of TILs through EMAP-II *in vitro*. Jurkat cells cocultured with tumour cells. FITC-labelled annexin-V assay (**A**) and caspase-3 activity (**B**) were performed to determine the level of apoptosis. (i) Jurkats alone; (ii) Jurkats cocultured with tumour cells; (iii) Jurkats cocultured with cells pretreated with TNF-*α*/IFN-*γ*; (iv) Jurkats cocultured with cells pretreated with TNF-*α*/IFN-*γ* in the presence of R2B2 blocking antibodies; (v) Jurkats cocultured with cells pretreated with TNF-*α*/IFN-*γ* in the presence of control IgG. The data shown are the averages of three experiments. The data shown are the averages of three experiments (mean±s.e.m.). ^**^ Indicates significance at *P*<0.05 in comparison to controls. (**C**) Concentration-dependent increase in caspase-3 activity in jurkats with decreasing concentrations of anti-EMAP-II antibody. Data are means; error bars represent s.e.m. (**D**) Caspase-3 activity in Jurkats over time. 1, Jurkats+HT29 pretreated with TNF-*α*/IFN-*γ* in normoxia; 2, Jurkats+HT29 pretreated with TNF-*α*/IFN-*γ* in hypoxia; 3, Jurkats+HT29 pretreated with TNF-*α*/IFN-*γ* and R2B2 antibodies in normoxia; 4, Jurkats+HT29 pretreated with TNF-*α*/IFN-*γ* and R2B2 antibodies in hypoxia. Data are presented as the mean±s.e.m. of at least three separate experiments.

The median percentage of apoptotic Jurkats cocultured with DLD-1 for 24 h in hypoxia is illustrated in Figure 3. Jurkats alone showed low levels of apoptosis under hypoxic conditions (Figure 3Ai). However, Jurkat cells displayed increased apoptosis when cultured with hypoxic tumour cells (*P*=0.03) (Figure 3Aii). Coculture of Jurkats with cytokine-treated tumour cells in hypoxia caused a significant increase in apoptosis in comparison to control (*P*<0.05) (Figure 3Aiii). To determine whether EMAP-II is indeed responsible for apoptosis of T-cells induced by hypoxia in CRC, we performed blocking experiments using R2B2 antibodies or purified normal rabbit IgG as a control. Blocking experiments using the anti-EMAP-II antibody in the coculture caused a marked reduction in the percentage of apoptotic Jurkat cells in hypoxia (Figure 3Aiv). Control IgG had no effect on hypoxia-induced lymphocyte apoptosis in cocultures (Figure 3Av). For HT29 cells, we have the same data (supplementary Figure 3). Flow cytometric analysis of apoptosis in Jurkat cells are given in supplementary Figure 3.

Caspase-3 activity was assayed in extracts of control Jurkats and Jurkats cocultured under a variety of conditions with tumour cells (Figure 3B). Jurkats cultured alone showed low levels of apoptosis in hypoxia. Coculture of Jurkats with HT29 cells for 4 h caused a significant increase in the activity of caspase-3 in hypoxia. Coculture with HT29 cells pretreated with TNF-*α*/IFN-*γ* induced a significant increase in caspase-3 activity in hypoxia (*P*<0.05). Addition of R2B2-blocking antibodies against EMAP-II to the coculture completely inhibited the generation of caspase-3 activity in Jurkat cells in hypoxia. Control IgG had no effect caspase-3 activity in these experiments.

Blocking effect of anti-EMAP-II antibody is concentration-dependent (Figure 3C). There was an increase in caspase-3 activity with decreasing concentrations of anti-EMAP-II antibody. Concentration of 10 *μ*g ml^−1^ completely inhibited caspase-3 activity. No effect was observed with the control one.

A significant increase in the activity of caspase-3 was observed with increasing time in hypoxia (Figure 3D). The marked increase in caspase-3-like activity may reflect an increase in the proportion of cells undergoing apoptosis in hypoxia.

### EMAP-II-induced lymphocyte apoptosis requires cell–cell contact

**Figure 4:**
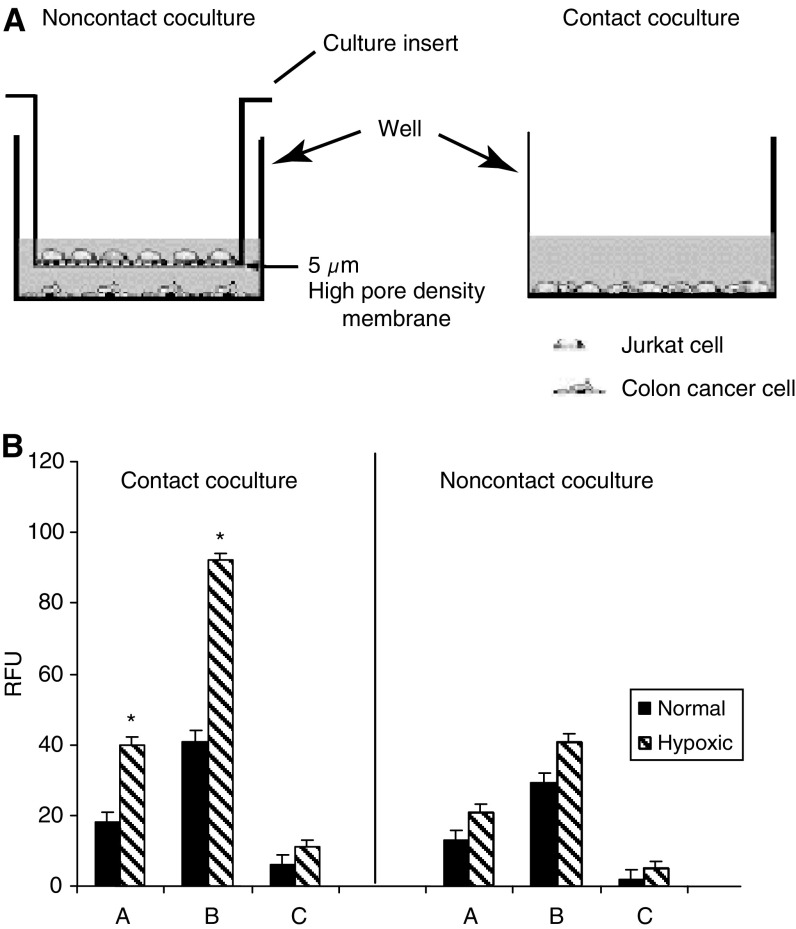
EMAP-II-Induced apoptosis of lymphocytes requires cell–cell contact. (**A**) Experimental design. Tumour cells and Jurkats were cocultured in contact and noncontact conditions. In the noncontact cocultures, tumour cells were plated at the bottom of a culture-plate well and Jurkats at the bottom of a culture insert. The culture insert was placed in the well and the cells were then incubated. In the contact cocultures, tumour cells and Jurkats were plated at the bottom of the same culture-plate well. (**B**) A=Jurkats were cultured with tumour cells, B=Jurkats+tumour cells pretreated with TNF-*α*/IFN-*γ*, C=Jurkats+tumour cells pretreated with TNF-*α*/IFN-*γ* and R2B2. Results represent the mean of three determinations±s.e.m. ^*^ Indicates significant deviations from control values, *P*<0.05.

To examine whether EMAP-II-induced lymphocyte apoptosis was mediated by direct cell–cell contact (Figure 4), apoptosis of TILs was analysed by separating the colorectal cancer cell lines and Jurkats as a model for T lymphocytes through a 5 *μ*m filter (Figure 4A). Contact coculture of Jurkats for 4 h with tumour cells either untreated or treated with TNF-*α*/IFN-*γ* caused a significant increase in caspase-3 activity compared to noncontact coculture (*P*<0.05) (Figure 4B). Addition of R2B2 blocking antibodies against EMAP-II to the contact coculture inhibited the activity of caspase-3. Thus, prevention of cell–cell contact reduced lymphocyte apoptosis, indicating that direct cell–cell contact is required for hypoxia-induced lymphocyte apoptosis.

## DISCUSSION

It is well known that hypoxia can lead to cellular injury and death, both through induction of apoptosis and via necrotic cell death (Chae *et al*, 2001). In the present study, we provide novel evidence for the participation of hypoxia in protecting tumours against the immune system, by showing that hypoxia can lead to death of tumour-infiltrating lymphocytes via an EMAP-II-dependent mechanism.

TILs have been shown to be capable of specifically eliminating tumour cells (Aebersold *et al*, 1991), but also to be rendered potentially nonfunctional due to tumour-induced immunosuppression (Finke *et al*, 1990). In a previous study, we hypothesised that EMAP-II might act in such an immunosuppressive role, by directly inducing apoptosis in mitogen-activated lymphocytes (Murray *et al*, 2004).

In this paper, we have used a number of techniques to demonstrate a possible link between EMAP-II expression, hypoxia (which we previously showed to enhance EMAP-II expression), and lymphocyte apoptosis *in vivo* and *in vitro*. Our data show that hypoxia is spatially associated with apoptosis in TILs in CRC. *In vivo*, we observed that hypoxic regions contain significant numbers of TILs demonstrating the presence of active caspase-3 and proteolytic cleavage of PARP. As caspase-3 is a critical mediator of apoptosis (Slee *et al*, 1999), it may be a potential marker for predicting apoptosis in TILs in colorectal cancer. Activation of caspase-3 has been linked to the proteolytic cleavage of cellular substrates including PARP (Nicholson and Thornberry, 1991).

In a previous study, we have shown that low oxygen levels can enhance EMAP-II release and conversion (Barnett *et al*, 2000). Hypoxia is known to upregulate the expression and release of matrix metalloproteinases and plasminogen activator-1 from tumour cells (Koong *et al*, 2000; Canning *et al*, 2001), and these enzymes could potentially be involved in EMAP-II processing at the cell surface. In this article, we could demonstrate *in vitro* that hypoxia leads to the cleavage of proEMAP-II and the subsequent release of mature EMAP-II protein from DLD-1 cells. However, barely detectable mature EMAP-II was detected in supernatants derived from hypoxic HT29 cells. This is consistent with previous publication (Murray *et al*, 2004). With this study, we are the first to analyse surface expression of EMAP-II in hypoxia. Analysis of EMAP-II surface expression in HT29 cells revealed significant difference between hypoxic and normal cells. We observed increase in EMAP-II surface expression in hypoxic DLD-1 cells.

In this article, we now demonstrate by immunohistochemistry a strong association between EMAP-II, active caspase-3, and cleaved PARP in TILs within tumours. Furthermore our study confirms an association between expression of EMAP-II and that of CA IX, a surrogate marker of hypoxia, in colorectal tissues. These findings are supported by the *in vitro* studies, which show that hypoxia alone induces only a small change in levels of apoptosis in Jurkat cells. However, increased levels of apoptosis were seen when colorectal cancer cells were cocultured with Jurkat cells in normoxia, and even higher levels in hypoxic cocultures. Pretreatment of colorectal cancer cells with cytokines again produced increased apoptosis of Jurkat cells in hypoxia. By preincubating tumour cells with blocking antibody, this phenomenon was demonstrated to be EMAP-II-dependent.

We also showed previously that EMAP-II treatment leads to activation of caspase-8 in lymphocytes, implicating a death receptor pathway (Murray *et al*, 2004). Data in the present study present further evidence in support of this hypothesis, in the form of caspase-3 activation and PARP cleavage. However, while it is clear that a caspase cascade probably plays a role, the nature of the death receptor is not clear.

In the *in vitro* studies, caspase-3 activity was low in normoxic noncontact cocultures, although Jurkat apoptosis was increased in these conditions compared to single cell type cultures, suggesting that more than one mechanism may be responsible for Jurkat cell death in this model. Levels of caspase-3 rose significantly in hypoxic conditions, a phenomenon which has been reported by others (Todor *et al*, 2002; Parikh *et al*, 2003). In our experiments, active caspase-3 was observed in Jurkat cells cocultured with HT29 pretreated with TNF-α/IFN-*γ* within 4 h of hypoxia treatment, with increasing activity over time in hypoxia. While cytokine pretreatment of tumour cells increased Jurkat cell death, the effect was always enhanced in hypoxic conditions. These data suggest that hypoxia plays an important role in the viability of lymphocytes within the tumour microenvironment.

Previous studies have shown that EMAP-II can induce apoptosis either through direct cell–cell contact, or by acting as a soluble mediator signalling cell death (Murray *et al*, 2004). Our current data, for both tumour cell lines, suggest that direct cell–cell contact is a more potent mechanism for induction of lymphocyte apoptosis in hypoxia. While there may be other mechanisms contributing to cell killing when there is contact, the equivalent effect of antibody blocking in contact and noncontact cultures suggest that the EMAP-II mechanism predominates.

In summary, we have reported that in colorectal cancer hypoxia stimulates apoptosis in TILs via an EMAP-II-dependent mechanism. It is well established that poor oxygenation of solid tumours is associated with poor prognosis, and it has been suggested that this may have less to do with direct effects of hypoxia on the efficacy of treatment modalities, and more to do with the development of resistant tumour cell clones under hypoxic conditions (Weinmann *et al*, 2004). Our study suggests that hypoxia induces apoptosis in TILs via an EMAP-II-dependent mechanism. This may represent yet another means by which the presence of hypoxia confers a growth advantage on tumour cells.

## Figures and Tables

**Table 1:** Relationship between CA IX expression and clinico-pathological features

**Variables**	**Total**	**1–25%**	**26–50%**	**51–75%**	**76–100%**	***P*-value^a^**
*Age tertile*						NS^b^
<61	20	6	5	6	3	
61.1–71.9	22	7	8	3	4	
⩾72	30	3	14	10	3	
						
*Type*						NS
Nonmucinous	66	15	23	18	10	
Mucinous	6	1	4	1		
						
*Site*						NS
Left colon	3		1	1	1	
Caecum	17	4	8	5		
Right colon	4	1	1	1	1	
Transverse, splenic	3		1		2	
Rectum	24	5	8	8	3	
Rectum-sigmoid	8	2	3	1	2	
Sigmoid	13	4	5	3	1	
						
*Size median*						NS
<Median	28	5	11	10	2	
⩾Median	44	11	16	9	8	
						
*Dukes' stage*						<0.01
A	18	10	3	4	1	
B	32	6	21	4	1	
C	22		3	11	8	
						
*Differentiation*						0.01
Moderate	4	1	1	1	1	
Well	62	14	25	18	5	
Poor	6	1	1	3	1	
						
*Metastasis*						0.01
Primary	72	16	27	19	10	
Secondary	13	2	5	4	2	
						
*Lymphatic metastasis*						0.01
At DX^c^	24	1	4	11	8	
During FU^d^	2	1	1			
No metastasis	46	14	22	8	2	
						
*Vascular metastasis*						0.05
<Median	37	6	16	9	6	
⩾Median	35	10	11	10	4	
						
*Lymph node metastasis*						0.02
No	43	14	20	8	1	
Yes	29	2	7	11	9	
						
*Death*						0.03
No	28	10	9	6	3	
Yes	44	6	18	13	7	
						
*Recurrence*						0.02
Yes	25	1	9	9	6	
No	42	14	17	8	3	
NA	5	1	1	2	1	
						
*Gender*						NS
Male	42	9	13	13	7	
Female	30	7	14	6	3	

a *P*-value by multivariate regression analysis.

b Not significant.

c At Dukes' C.

d Nodal recurrence.

**Table 2:** Relationship between cleaved PARP in TILs and clinico-pathological features

**Variables**	**Total**	**1–25%**	**26–50%**	**51–75%**	**76–100%**	***P*-value^a^**
*Age tertile*						NS^b^
<61	20	13	1	4	2	
61.1–71.9	22	7	5	9	1	
⩾72	30	9	8	12	1	
						
*Type*						NS
Nonmucinous	66	27	14	21	4	
Mucinous	6	2		4		
						
*Site*						NS
Left colon	3	1		2		
Caecum	17	5	4	6	2	
Right colon	4	2		2		
Transverse, splenic	3	2	1			
Rectum	24	9	2	13		
Rectum-sigmoid	8	4	2	2		
Sigmoid	13	6	5	2		
						
*Size median*						NS
<Median	28	10	7	9	2	
⩾Median	44	19	7	16	2	
						
*Dukes' stage*						0.02
A	18	10	3	4	1	
B	32	12	10	9	1	
C	22	7	1	12	2	
						
*Differentiation*						NS
Moderate	4	2	1	1		
Well	62	25	12	22	3	
Poor	6	2	1	2	1	
						
*Metastasis*						NS
Primary	72	29	14	25	4	
Secondary	13	1	4	6	2	
						
*Lymphatic metastasis*						0.04
At DX^c^	24	9		13	2	
During FU^d^	2		1	1		
No metastasis	46	20	13	11	2	
						
*Vascular metastasis*						NS
<Median	37	16	7	12	2	
⩾Median	35	13	7	13	2	
						
*Lymph node metastasis*						NS
No	43	16	5	13	9	
Yes	29	13	9	2	5	
						
*Death*						NS
No	28	12	6	10		
Yes	44	17	8	15	4	
						
*Recurrence*						NS
Yes	25	9	10	6		
No	42	19	4	14	5	
NA	5	1		4		
						
*Gender*						NS
Male	42	15	7	17	3	
Female	30	14	7	8	1	

a *P*-value by multivariate regression analysis.

b Not significant.

c At Dukes' C.

d Nodal recurrence.

**Table 3:** Relationship between EMAP-II expression and clinico-pathological features

**Variables**	**Total**	**1–25%**	**26–50%**	**51–75%**	**76–100%**	***P*-value^a^**
*Age tertile*						NS^b^
<61	20	3	2	4	11	
61.1–71.9	22	1	6	5	10	
⩾72	30	3	6	7	14	
						
*Type*						NS
Nonmucinous	66	7	13	15	31	
Mucinous	6		1	1	4	
						
*Site*						NS
Left colon	3			2	1	
Caecum	17	2	5	3	7	
Right colon	4				4	
Transverse, splenic	3				3	
Rectum	24	3	3	6	12	
Rectum-sigmoid	8		2	1	5	
Sigmoid	13	2	4	6	1	
						
*Size median*						NS
<Median	28	5	4	5	14	
⩾Median	44	2	10	12	20	
						
*Dukes' stage*						<0.01
A	18	2	4	3	9	
B	32	4	7	8	13	
C	22	1	3	5	13	
						
*Differentiation*						NS
Moderate	4	1			3	
Well	62	6	12	15	29	
Poor	6		2	1	3	
						
*Metastasis*						0.05
Primary	72	7	14	16	35	
Secondary	13	1	1	4	7	
						
*Lymphatic metastasis*						0.05
At DX^c^	24	1	4	6	13	
During FU^d^	2		2			
No metastasis	46	6	10	8	22	
						
*Vascular metastasis*						0.01
<Median	37	5	6	9	17	
⩾Median	35	2	8	7	18	
						
*Lymph node metastasis*						<0.01
No	43	6	9	8	20	
Yes	29	1	5	8	15	
						
*Death*						0.03
No	28	3	5	8	12	
Yes	44	4	9	8	23	
						
*Recurrence*						0.01
Yes	25	1	5	7	12	
No	42	6	9	7	20	
NA	5			2	3	
						
*Gender*						NS
Male	42	3	10	7	22	
Female	30	4	4	9	13	

a *P*-value by multivariate regression analysis.

b Not significant.

c At Dukes' C.

d Nodal recurrence.
