# Supplementary material for: EMAP-II-dependent lymphocyte killing is associated with hypoxia in colorectal cancer
Source: Br J Cancer. 2006 Aug 22;95(6):735–43. doi: 10.1038/sj.bjc.6603299 (PMC2360520; doi:10.1038/sj.bjc.6603299)
Supplement: Supplementary Table 1 [file 95-6603299x1.doc]

| **Variable** | **N (%)** |
| --- | --- |
| **Age tertile** |  |
| <61 | 20 (28) |
| 61-71.9 | 22 (30) |
| >72 | 30 (42) |
| **Age median** |  |
| < median | 34 (47) |
| ≥ median | 38 (53) |
| **Gender** |  |
| Male | 42 (59) |
| Female | 30 (41) |
| **Type** |  |
| Non-mucinous | 66 (91) |
| Mucinous | 6 (9) |
| **Site** |  |
| Left colon | 3 (4.5) |
| Caecum | 17 (24) |
| Right colon | 4 (5) |
| Transverse, splenic | 3 (4.5) |
| Rectum | 24 (33) |
| Rectum-sigmoid | 8 (11) |
| Sigmid | 13 (18) |
| **Dukes’ stage** |  |
| A | 18 (25) |
| B | 32 (44) |
| C | 22 (31) |
| **Differentiation** |  |
| Moderately | 4 (5) |
| Well | 62 (86) |
| Poor | 6 (9) |
| **Metastasis** |  |
| Primary | 72 |
| Secondary | 13 (18) |
| **Lymphatic metastasis** |  |
| At DX | 24 (33) |
| During FU | 2 (4) |
| No metastasis | 46 (63) |
| **Vascular metastasis** |  |
| <median | 37 (51) |
| ≥median | 35 (49) |
| **LN metastasis** |  |
| No | 43 (59) |
| Yes | 29 (41) |
| **Death** |  |
| No | 28 (38) |
| Yes | 44 (62) |
| **Recurrence** |  |
| Yes | 25 (34) |
| No | 42 (58) |
| n/a | 5 (8) |
| **Size median** |  |
| < median | 28 (38) |
| ≥ median | 44 (62) |

**Supplementary Table 1: Patient and tumour characteristics in colorectal cancer.**
