# Supplementary material for: EMAP-II-dependent lymphocyte killing is associated with hypoxia in colorectal cancer
Source: Br J Cancer. 2006 Aug 22;95(6):735–43. doi: 10.1038/sj.bjc.6603299 (PMC2360520; doi:10.1038/sj.bjc.6603299)
Supplement: Supplementary Table 2 [file 95-6603299x2.doc]

| **Variables** | **Total** | **1-25%** | **26-50%** | **51-75%** | **76-100%** | ***p* value*^a^*** |
| --- | --- | --- | --- | --- | --- | --- |
| **Age tertile** | 72 |  |  |  |  | **NS*^b^*** |
| <61 | 20 | 9 | 5 | 4 | 2 |  |
| 61.1-71.9 | 22 | 8 | 6 | 8 | 2 |  |
| >72 | 30 | 12 | 7 | 6 | 1 |  |
| **Age median** |  |  |  |  |  | **NS** |
| <median | 34 | 12 | 9 | 9 | 4 |  |
| ≥median | 38 | 17 | 9 | 10 | 2 |  |
| **Type** |  |  |  |  |  | **NS** |
| Non-mucinous | 66 | 27 | 18 | 16 | 5 |  |
| Mucinous | 6 | 2 |  | 3 | 1 |  |
| **Site** |  |  |  |  |  | **NS** |
| Left colon | 3 | 2 | 1 |  |  |  |
| Caecum | 17 | 7 | 3 | 6 | 1 |  |
| Right colon | 4 | 1 | 2 | 1 |  |  |
| Transverse, splenic | 3 | 1 |  | 1 | 1 |  |
| Rectum | 24 | 8 | 7 | 7 | 2 |  |
| Rectum-sigmoid | 8 | 2 | 4 | 1 | 1 |  |
| Sigmoid | 13 | 8 | 1 | 3 | 1 |  |
| **Size median** |  |  |  |  |  | **NS** |
| <median | 28 | 12 | 7 | 6 | 3 |  |
| ≥median | 44 | 17 | 11 | 13 | 3 |  |
| **Dukes’ stage** |  |  |  |  |  | **0.02** |
| A | 18 | 5 | 6 | 5 | 2 |  |
| B | 32 | 15 | 7 | 9 | 1 |  |
| C | 22 | 9 | 5 | 5 | 3 |  |
| **Differentiation** |  |  |  |  |  | **NS** |
| Moderate | 4 |  | 3 | 1 |  |  |
| Well | 62 | 26 | 14 | 17 | 5 |  |
| Poor | 6 | 3 | 1 | 1 | 1 |  |
| **Metastasis** |  |  |  |  |  | **NS** |
| Primary | 72 | 30 | 18 | 18 | 6 |  |
| Secondary | 13 | 2 | 2 | 9 |  |  |
| **Lymphatic metastasis** |  |  |  |  |  | **NS** |
| At DX***^c^*** | 24 | 11 | 4 | 6 | 3 |  |
| During FU*^d^* | 2 |  | 1 | 1 |  |  |
| No metastasis | 46 | 18 | 13 | 12 | 3 |  |
| **Vascular metastasis** |  |  |  |  |  | **NS** |
| <median | 37 | 17 | 8 | 8 | 4 |  |
| ≥median | 35 | 12 | 10 | 11 | 2 |  |
| **LN metastasis** |  |  |  |  |  | **NS** |
| No | 43 | 16 | 4 | 13 | 10 |  |
| Yes | 29 | 13 | 14 | 2 |  |  |
| **Death** |  |  |  |  |  | **NS** |
| No | 28 | 8 | 9 | 9 | 2 |  |
| Yes | 44 | 21 | 9 | 10 | 4 |  |
| **Recurrence** |  |  |  |  |  | **NS** |
| Yes | 25 | 12 | 4 | 7 | 2 |  |
| No | 42 | 15 | 12 | 11 | 4 |  |
| n/a | 5 | 2 | 2 | 1 |  |  |
| **Gender** |  |  |  |  |  | **NS** |
| Male | 42 | 13 | 13 | 13 | 3 |  |
| Female | 30 | 16 | 5 | 6 | 3 |  |

**Supplementary Table 2: Relationship between active caspase-3 in TIL and clinico-pathological features. *^a^* *p* value by multivariate regression analysis; *^b^*not significant; *^c^* at Dukes’ C; *^d^* nodal recurrence.**
