# Supplementary figures and images for: EMAP-II-dependent lymphocyte killing is associated with hypoxia in colorectal cancer
Source: Br J Cancer. 2006 Aug 22;95(6):735–43. doi: 10.1038/sj.bjc.6603299 (PMC2360520; doi:10.1038/sj.bjc.6603299)

**
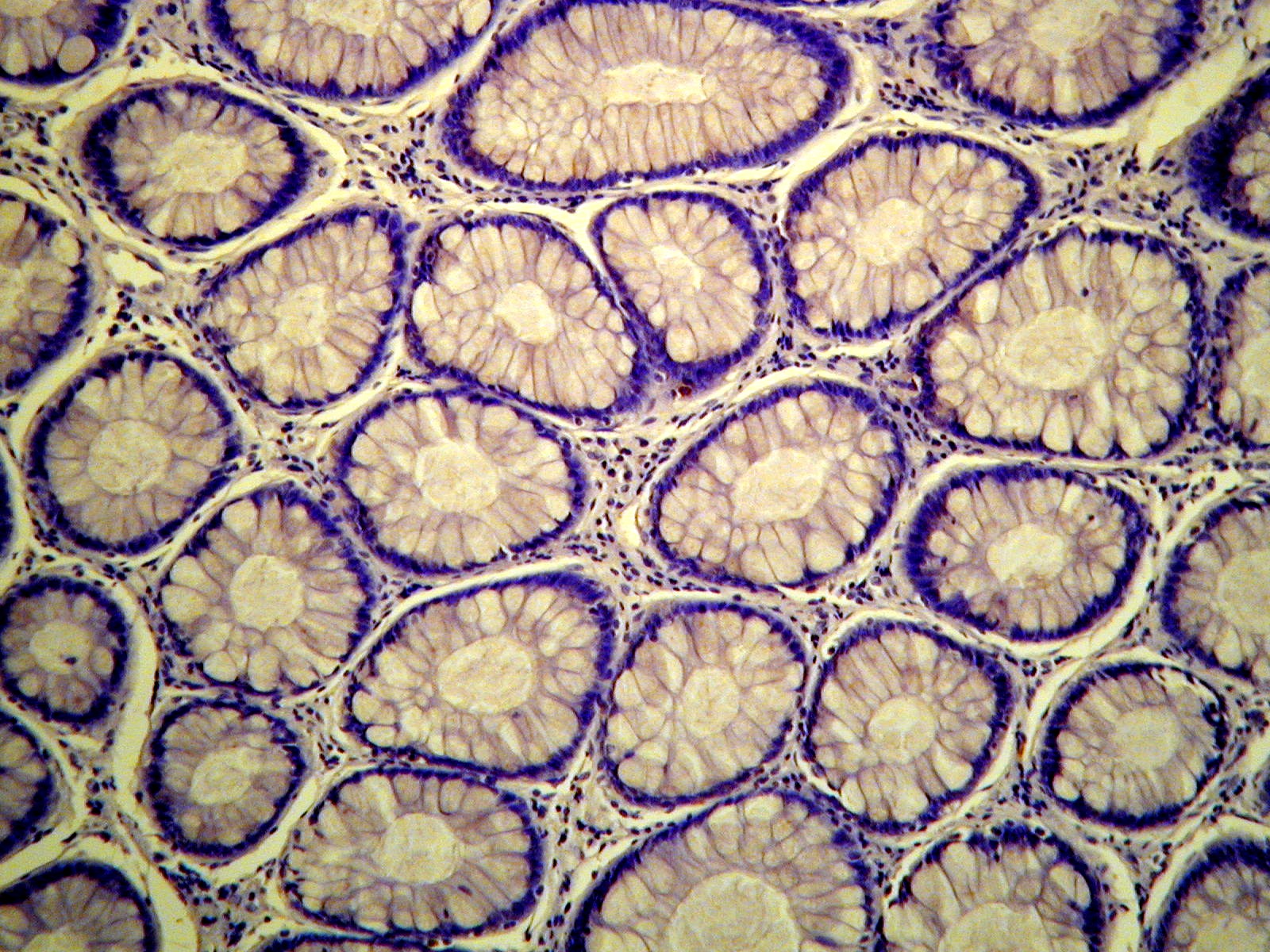
**

**Supplementary Figure 1: TILs in normal colon.** One apoptotic lymphocyte is detected.

Supplement: Supplementary Figure 1 [file 95-6603299x3.doc]
