# Supplementary material for: EMAP-II-dependent lymphocyte killing is associated with hypoxia in colorectal cancer
Source: Br J Cancer. 2006 Aug 22;95(6):735–43. doi: 10.1038/sj.bjc.6603299 (PMC2360520; doi:10.1038/sj.bjc.6603299)
Supplement: Supplementary Figure 2 [file 95-6603299x4.doc]

**Supplementary Figure 2: HT29 cells releases barely detectable mature EMAP-II *in vitro***. ELISA of conditioned medium from HT29 cells for soluble EMAP-II in normal and hypoxic conditions. Data represent mean of three determinations ± S.E.M
