# Supplementary material for: EMAP-II-dependent lymphocyte killing is associated with hypoxia in colorectal cancer
Source: Br J Cancer. 2006 Aug 22;95(6):735–43. doi: 10.1038/sj.bjc.6603299 (PMC2360520; doi:10.1038/sj.bjc.6603299)
Supplement: Supplementary Figure 3 [file 95-6603299x5.doc]

**Supplementary Figure 3A:** **FITC-labeled annexin-V assay was performed to determine** **the level of apoptosis**. **(i)** Jurkats alone; **(ii)** Jurkats cocultured with tumour cells; **(iii)** Jurkats cocultured with cells pretreated with TNF-α/IFN-γ; **(iv)** Jurkats cocultured with cells pretreated with TNF-α/IFN-γ in the presence of R2B2 blocking antibodies; **(v)** Jurkats cocultured with cells pretreated with TNF-α/IFN-γ in the presence of control IgG. The data shown are the averages of three experiments. The data shown are the averages of three experiments (mean ± SEM). ** Indicates significance at *p*<0.05 in comparison to controls.


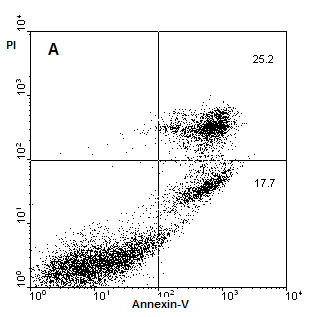


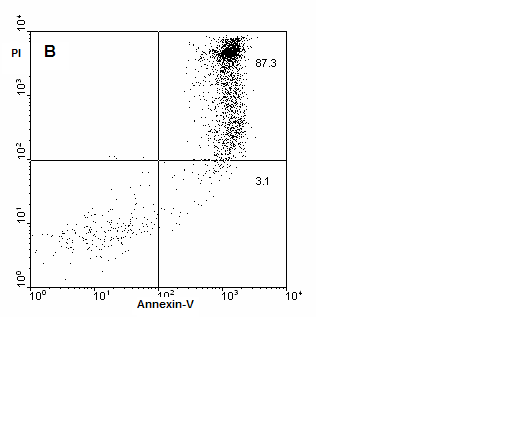
 **
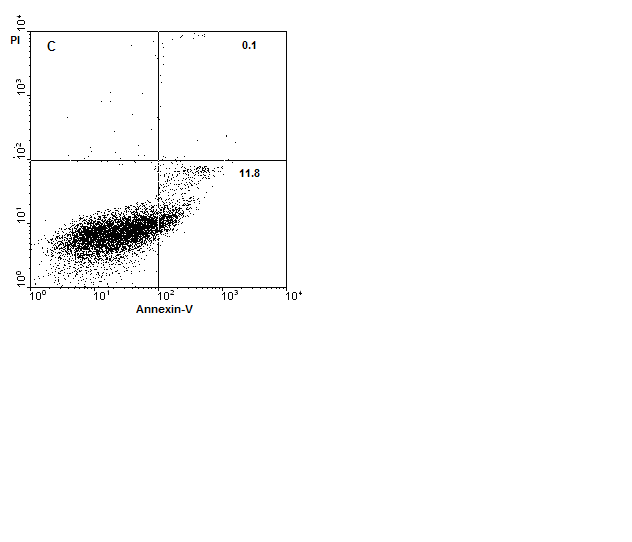
**

**Supplementary Figure 3B: Hypoxia-inducing apoptosis in Jurkat cells is EMAP-II dependent.** **(A)** Jurkat cells were cultured with untreated tumour cells in hypoxia; **(B)** Jurkats cocultured with cells pretreated with TNF-α/IFN-γ in hypoxia; **(C)** Jurkats cocultured with cells pretreated with TNF-α/IFN-γ in the presence of R2B2 blocking antibodies under hypoxic conditions.
